# Distinct Extracellular Matrix Protein Signatures of Cortical and Cancellous Bone Allografts Following Processing for Clinical Use

**DOI:** 10.3390/cells15090842

**Published:** 2026-05-04

**Authors:** Adrian Lendvai, Hans Peter Weitzenböck, Christian Klein, Christoph Wiesner, Rita Seeboeck, Barbara Entler, Benjamin Neuditschko, Franz Herzog, Michael Matzner, Monika Pichler, Andrea De Luna, Stefan Nehrer, Harald Hundsberger

**Affiliations:** 1 Department of Life Sciences, IMC University of Applied Sciences, 3500 Krems, Austria; adrian.lendvai@imc.ac.at (A.L.); hans.weitzenboeck@imc.ac.at (H.P.W.); christian.klein@imc.ac.at (C.K.); christoph.wiesner@imc.ac.at (C.W.); rita.seeboeck@imc.ac.at (R.S.); barbara.entler@imc.ac.at (B.E.); 2 Center for Regenerative Medicine, University for Continuing Education Krems, 3500 Krems, Austria; andrea.de-luna@donau-uni.ac.at (A.D.L.); stefan.nehrer@donau-uni.ac.at (S.N.); 3 Institute of Clinical Pathology and Molecular Pathology of the Lower Austria Central Region (University Hospital St. Pölten), Karl Landsteiner University of Health Sciences, 3500 Krems, Austria; 4 Institute Krems Bioanalytics, IMC University of Applied Sciences, 3500 Krems, Austria; benjamin.neuditschko@imc.ac.at (B.N.); franz.herzog@imc.ac.at (F.H.); 5 Cells + Tissuebank Austria Gemeinnützige GmbH, 3500 Krems, Austria; michael.matzner@meduniwien.ac.at (M.M.); m.pichler@ctba.at (M.P.); 6 Department of Orthopedics and Traumatology, Medical University of Vienna, 1090 Vienna, Austria; 7 Department of Dermatology, University Hospital of the Paracelsus Medical University, 90419 Nürnberg, Germany

**Keywords:** demineralized bone matrix, extracellular matrix, bone allografts, cortical bone, cancellous bone, proteomics, mass spectrometry, gamma irradiation, bone remodeling, protein preservation

## Abstract

**Highlights:**

**What are the main findings?**

- DIA-based proteomics identified distinct extractable ECM-associated protein signatures in processed cortical and cancellous bone allografts.
- Across the more extensively processed allograft products, fewer strongly differential proteins were observed, but source-associated differences remained detectable across all product stages.

**What are the implications of the main findings?**

- Cancellous allografts were relatively enriched in coagulation-, inflammatory-, and immune-associated proteins, whereas cortical-derived products retained more structural and matrix-organization-associated proteins.
- Proteomic profiling provides a molecular framework for future donor-resolved and functionally validated evaluation of DBM allografts.

**Abstract:**

Demineralized bone matrices (DBMs) are widely used in bone replacement therapy. Bone tissue of either cancellous or cortical origin is decellularized, demineralized, and sterilized during processing, while retaining portions of native organic extracellular matrix (ECM) proteins that regulate cell–matrix interactions during bone repair. The ECM largely accounts for the distinct functions of cortical and cancellous bone. Differences in three-dimensional architecture and matrix density between cancellous and cortical bone may therefore affect ECM proteome signatures and the resulting cellular microenvironment. In this study, ECM proteins were extracted from processed cancellous and cortical allografts at multiple processing steps and analyzed by quantitative mass spectrometry. We identified distinct extractable proteome signatures associated with bone metabolic functions. Cancellous grafts were relatively enriched in proteins associated with inflammatory, coagulative, and immune-related processes, whereas cortical grafts showed higher abundance of structural and matrix-organization-associated proteins. More extensively processed product formats showed fewer significant protein differences between the cortical and cancellous bone type. Within the limitations of pooled donor material and absent functional validation, these findings provide a proteomic framework for future characterization and evaluation of DBM-based allograft products.

## 1. Introduction

Bone is a highly specialized connective tissue with a hierarchical structure that serves mechanical and metabolic functions. At the macroscopic level, two major structural types can be distinguished: cortical and cancellous bone [1]. Cortical bone forms the dense outer shell of long bones, characterized by low porosity, high mechanical strength, and slow remodeling characteristics. Cancellous bone is predominantly located in epiphyseal regions and vertebral bodies, where its highly porous architecture and large surface area allow for dynamic metabolic exchange [2,3,4]. The distinct functional roles of cortical and cancellous bone are largely determined by the composition and organization of the extracellular matrix (ECM). Beyond providing mechanical stability, the bone ECM regulates cellular activity, growth factor signaling, and immune responses, thereby controlling site-specific bone turnover and remodeling [5,6,7,8,9,10,11,12].

These insights highlight the importance of ECM preservation in bone repair strategies, particularly in the context of allograft-based therapies. Human bone allografts are widely used to repair skeletal defects due to their osteoconductive and osteoinductive properties. However, to ensure safety and reduce immunogenicity, grafts undergo decellularization and sterilization. Demineralization is performed to enhance osteoinductive properties resulting in demineralized bone matrices (DBMs). DBMs are clinically applied as powders, fibers or pastes/putties, which are frequently preloaded into syringes to enable standardized handling and controlled delivery [13,14]. While mineral components are largely removed, portions of the organic extracellular matrix proteome remain detectable. Nevertheless, processing steps can compromise ECM integrity, and methods such as gamma irradiation have been shown to reduce osteoinductive potential by damaging sensitive matrix-associated proteins [13,15,16,17]. Given the inherent biological differences between cortical and cancellous bone, it is plausible that these tissues retain distinct ECM protein profiles even after processing for clinical use. Paleoproteomic studies suggest intrinsic differences in protein stability between cortical and cancellous bone, supporting the idea that these compartments may differ in ECM protein preservation [18]. Accordingly, cortical grafts may preferentially retain proteins involved in matrix organization and long-term structural stability, whereas cancellous grafts may exhibit higher relative levels of marrow- or plasma-associated proteins linked to early inflammatory and fibrovascular responses during graft incorporation [19,20,21]. Despite the widespread clinical use of DBMs, it remains unclear whether cortical- and cancellous-derived allografts retain distinct ECM proteomic signatures following standardized processing for clinical applications.

We therefore hypothesize that processed cortical and cancellous allografts reveal distinct proteomic patterns reflecting their biological roles. Specifically, cortical grafts are expected to be enriched in proteins supporting ECM organization and ossification, whereas cancellous grafts are expected to be enriched in proteins related to coagulation, immune modulation, and early regenerative responses. To test this hypothesis, we applied quantitative proteomics to compare extractable ECM protein profiles of standardized cortical and cancellous allograft products across defined processing steps. This work aims to provide a proteomic basis for future studies on characterization, quality assessment, and indication-oriented evaluation of DBM-based graft materials.

## 2. Materials and Methods

### 2.1. Bone Graft Preparation

Cortical and cancellous allograft samples were provided by Cells + Tissuebank Austria gemeinnützige GmbH, Krems, Austria (Austrian commercial register no. FN 243394h, Regional Court Krems). As this study used human allograft tissue and no cell lines, a cell-line database entry and accession number are not applicable. Cortical material was derived from the femoral diaphysis, whereas cancellous material was derived from the femoral head. A schematic illustration of the anatomical source regions is provided in Figure S1. Native bone was processed into graft material following the Allotec^®^ purification procedure in compliance with the tissue bank’s internal regulatory procedures. This included mechanical cleaning (cutting, sawing, centrifugation), chemical cleaning (ultrasonic bath with water-for-injection (WFI), treatment with diethyl ether, ethanol, and hydrogen peroxide), followed by lyophilization, demineralization, heat treatment, rehydration, Putty formulation, and sterilization. Demineralization was performed using 0.6 M HCl. Thermal treatment was performed at temperatures below 65 °C. Heat treatment, rehydration, and Putty formulation were performed according to the validated national and international manufacturing protocols of the tissue bank and are therefore not disclosed in detail (European Directorate for the Quality of Medicines & HealthCare; Gewebebankenverordnung) [22,23]. These procedures are essential for creating paste-like and moldable consistencies. Irradiation was performed by gamma irradiation at 25–35 kGy according to the manufacturer’s validated sterilization protocol. The grafts were categorized into three experimental groups reflecting increased processing: (1) demineralized bone granules (DBG), (2) wet heat-treated DBG, referred to as Putty heat-treated (PHT), and (3) gamma-irradiated PHT, referred to as Putty gamma-irradiated (PGI). Each group was prepared from both cortical and cancellous allograft products, resulting in a total of six conditions. The analyzed materials originated from pooled donor manufacturing material. For cancellous material, donor pools comprised 5000 (DBG), 2300 (PHT), and 2000 (PGI) donors. For cortical material, the corresponding pools comprised 451 (DBG), 64 (PHT), and 4 (PGI) donors. Measurements represent technical replicates of pooled material rather than donor-level biological replicates. Donor-level variables such as sex and age were not available for stratified analysis.

### 2.2. Protein Extraction and Quantification

Proteins were extracted from 50 mg of the bone grafts using 8 M urea (Carl Roth GmbH + Co. KG; Karlsruhe, Germany; 3941.3) in 20 mM ammonium bicarbonate (ABC) buffer (pH 8.0) (Merck KGaA; Vienna, Austria; 09830-500G) at room temperature for 24 h under constant agitation. Following extraction, lysates were isolated by centrifugation at 8000× *g* for 1 min, followed by 12,000× *g* for 1 min. The supernatants were diluted to 1 M urea in 20 mM ABC buffer, and the total protein concentration was determined using the Pierce™ BCA Protein Assay Kit (Cell Signaling Technology Europe; Leiden, The Netherlands; 7780S) employing a bovine serum albumin (BSA) standard curve ranging from 0 to 0.5 µg/µL in six dilutions.

### 2.3. Protein Digest

Equal amounts of protein (6 µg per sample) were enzymatically digested using the iST Sample Preparation Kit, 96× (PreOmics GmbH; Planegg, Germany; P.O.00027) according to the manufacturer’s protocol. The peptides were eluted twice with 90 µL elution buffer, which differed from the standard protocol. Accordingly, 180 µL of the eluted peptides were transferred into High-Performance Liquid Chromatography (HPLC) vials, followed by vacuum drying at 45 °C for approximately 4 h (Eppendorf; Vienna, Austria; Concentrator plus; Type 5305). The samples were stored at −80 °C.

### 2.4. High-Performance Liquid Chromatography with Mass Spectrometry (HPLC–MS)

Peptide measurements were carried out on an Ultimate 3000 RSLCnano system coupled to an Orbitrap Eclipse Tribrid mass spectrometer (Thermo Fisher Scientific; Vienna, Austria). Before analysis, dried peptide samples were dissolved in 12 µL of a solution containing LC-MS-grade water (Fisher Scientific; Vienna, Austria; W/0112/17), 2% acetonitrile (ACN) (Fisher Scientific; Vienna, Austria; A/0638/17), and 0.1% formic acid (Carl Roth GmbH + Co. KG; Karlsruhe, Germany; 1EHK.1). For each run, 2 µL of sample were loaded onto a PepMap RSLC EASY-Spray analytical column (C18, 2 µm, 100 Å, 75 µm × 50 cm; Thermo Fisher Scientific; Vienna, Austria; ES903). Chromatographic separation was performed at a flow rate of 300 nL/min using a linear gradient from 2% to 35% mobile phase B (2% H_2_O, 98% ACN, 0.1% FA) over 60 min, corresponding to a total run time of 80 min. Mass spectrometric acquisition was performed in positive-ion data-independent acquisition (DIA) mode using the FAIMS Pro interface with a compensation voltage of −45 V. Full MS scans were recorded from *m*/*z* 350 to 1400 at a resolution of 120,000 at *m*/*z* 200. DIA scans covered a precursor range of *m*/*z* 400–1000 using 14 *m*/*z* isolation windows with 1 *m*/*z* overlap, resulting in 43 sequential DIA windows. Fragmentation was induced by higher-energy collisional dissociation (HCD) at 30% normalized collision energy (NCE), and fragment ions were detected in the Orbitrap at a resolution of 30,000 at *m*/*z* 200. To increase proteome depth, a pooled sample generated from all study samples was additionally analyzed by gas-phase fractionation (GPF). For this purpose, the pooled material was measured in six consecutive injections using narrower precursor ranges of 100 *m*/*z* each (400–500, 500–600, 600–700, 700–800, 800–900, and 900–1000 *m*/*z*). In these GPF runs, DIA windows of 4 *m*/*z* with 2 *m*/*z* overlap were applied. Protein identification and quantification were performed with DIA-NN version 18.1.1. The GPF runs were first searched against the human UniProt database (release 10.2021; 20,386 entries), and the resulting identifications were used to generate a spectral library. The individual study samples were then analyzed against this spectral library in combination with the human FASTA database to maximize protein identification coverage. The mass spectrometry proteomics data have been deposited to the ProteomeXchange Consortium via the PRIDE partner repository under dataset identifier PXD075611.

### 2.5. Proteomic Data Analysis

HPLC–MS protein data were analyzed in R (Posit Software; version 2024.09.0+375). Protein intensities, treated as missing if not detected, were log_2_-transformed after adding a pseudocount (0.1 × 1st percentile of all non-zero intensities). Proteins were retained if they were detected in at least one processing step-by-bone-type subset fulfilling 4 of 6 valid sample HPLC–MS measurements per condition. No global detection or variance filtering was applied. RDA assessed proteome structure across bone types and steps, imputing missing data with Perseus-style Gaussian imputation. Differential abundance was analyzed per step with Limma, on log_2_ intensities without imputation. Only proteins with at least two observed values per group were included in the statistical testing, using FDR < 0.05 and log_2_FC > 1. The results were visualized with volcano plots. Protein functions were annotated with STRING Gene Ontology (GO) (Biological Process) analyses (version 12.0) [24]. The protein panels were visualized with heat maps and projected onto the initial RDA space. All scripts are provided in the Supplementary Materials.

## 3. Results

### 3.1. Proteomic Profiling Reveals Source- and Process-Dependent Clustering of Bone Allografts

**Figure 1:**
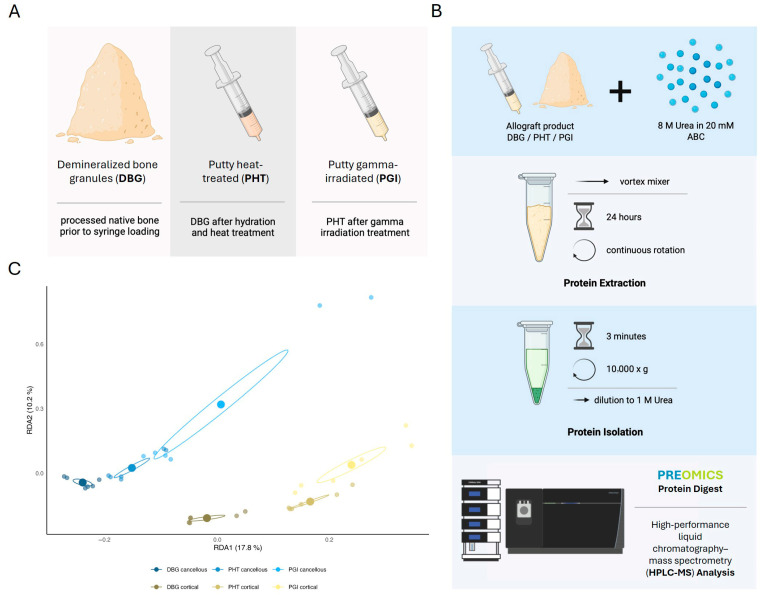
**Experimental workflow and proteomic differentiation of DBG, PHT, and PGI samples.** (**A**) Overview of the three allograft material types: demineralized bone granules (DBG), Putty heat-treated (PHT), and Putty gamma-irradiated (PGI). (**B**) Workflow for protein extraction and HPLC–MS analysis in DIA mode. (**C**) Redundancy analysis (RDA) of HPLC–MS protein abundance data. DBG, PHT, and PGI samples of each bone type form distinct clusters. Ellipses represent 20% confidence intervals for group variances, which are used for visual purposes only. Created with BioRender.com. Redundancy analysis (RDA) of the identified proteins revealed clear differences between cortical and cancellous bone matrices, as well as between processing methods (**C**). The ellipses represent the 20% confidence intervals for group variances, which are provided for visual purposes only. Cortical and cancellous samples formed separate groups along RDA1, while the processing stages (DBG, PHT, PGI) were distributed along RDA2; 17.8% of the variation in the ECM protein composition can be explained by bone type (RDA1), and 10.2% by the processing step (RDA2). For each bone type, DBG samples were followed by PHT and PGI samples. These results show separable clustering by bone type and the processing step, affecting the variation in the extractable proteome.

Protein extracts from DBG, PHT, and PGI samples were analyzed using HPLC–MS. The workflow involved protein solubilization in 8 M urea for 24 h, and enzymatic digestion before HPLC–MS analysis (Figure 1A,B). The solubilized bone proteins were analyzed by tandem mass spectrometry using a DIA workflow. The proteins were identified and quantitatively profiled using a spectral library generated by gas-phase fractionation (GPF). Across all samples, 1479 protein groups were identified. Protein intensities ranged from ~3 × 10^−2^ to ~8 × 10^7^ in MS2-based label-free quantification (LFQ). The number of protein identifications and intensity distributions for the individual sample types (DBG, PHT, and PGI) are summarized in the Supplementary Data.

### 3.2. Abundant Extracellular Matrix Proteins Are Detectable Across All Processing Stages

**Figure 2:**
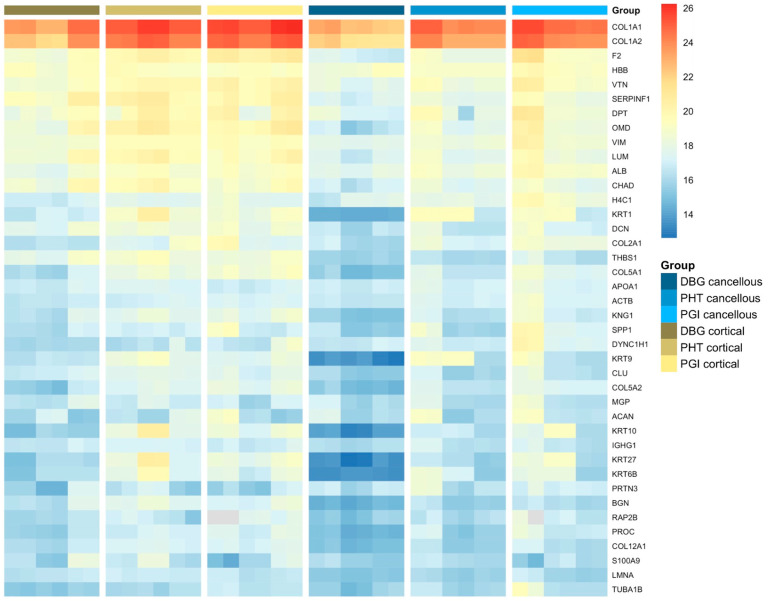
**Heatmap of the 40 most abundant proteins detected across cortical and cancellous allograft samples under different processing conditions (DBG, PHT, PGI).** Protein ranking was based on the mean label-free quantification (LFQ) intensity calculated from technical HPLC–MS replicates of pooled material for each analytical condition. For visualization, LFQ values of the individual technical replicates are shown. Distinct abundance patterns were observed among allograft types, with cortical samples generally showing higher and more uniform signal intensities for major extracellular matrix (ECM) proteins than cancellous samples. Color scale indicates relative LFQ intensity from low to high.

A heatmap of the 40 most abundant proteins displayed distinct abundance patterns across all allograft samples. Protein abundance ranking was based on mean LFQ values calculated from technical HPLC–MS replicates of pooled donor material for each analytical condition. For visualization, LFQ values of all individual replicates are shown for their respective samples (Figure 2). Type I collagen chains (COL1A1 and COL1A2) showed the highest overall intensities in all groups. Several extracellular matrix-associated proteins, including decorin (DCN), biglycan (BGN), vitronectin (VTN), lumican (LUM), and thrombospondin-1 (THBS1), were consistently detected. Cortical samples exhibited higher and more uniform signal intensities for major matrix proteins compared to cancellous samples. Cancellous bone showed lower overall abundance and greater variability. Protein abundance patterns differed across DBG, PHT, and PGI samples, with more homogeneous intensity distributions observed after processing. To put the detected ECM proteins into context within established phases of bone repair, proteins were categorized according to their reported biological roles (Table 1) [25,26].

### 3.3. Differential Protein Enrichment Indicates Distinct Functional Profiles

**Figure 3:**
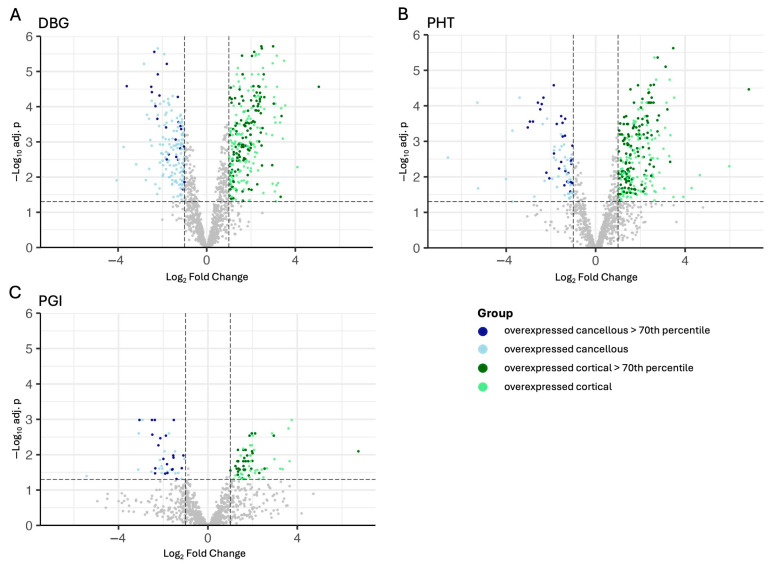
**Volcano plot analysis of protein abundance differences between cortical and cancellous allografts under different processing conditions.** Volcano plots display log2 fold-change (x-axis) versus −log10 adjusted p-value (y-axis) for all quantified proteins in (**A**) DBG, (**B**) PHT, and (**C**) PGI. Proteins with higher abundance in cancellous samples are shown in blue, and those with higher abundance in cortical samples are shown in green. Proteins with LFQ intensities above the 70th percentile are highlighted in darker shades to emphasize highly abundant and robustly detected proteins. Dashed vertical lines indicate the fold-change threshold (±1 log2FC), and the horizontal dashed line marks the significance threshold (−log10 of FDR 0.05) used for differential-abundance testing.

Volcano plot analysis revealed distinct protein abundance patterns between cortical and cancellous allografts under the different processing conditions (Figure 3A–C). For each product format, cortical and cancellous samples were compared separately: DBG (Figure 3A), PHT (Figure 3B), and PGI (Figure 3C). Proteins with higher abundance in cortical samples are shown in green, whereas proteins with higher abundance in cancellous samples are shown in blue. Proteins with LFQ values above the 70th percentile are highlighted in darker shades to indicate high abundance and robust detection. Overall, DBG and PHT showed broader distributions of significant cortical–cancellous differences, whereas PGI displayed fewer strongly differential proteins (Figure 3C).

Representative significantly differential proteins are compiled in Table S1. In DBG, cortical-associated proteins included BGN, DCN, OMD, MMP2, MMP14, THBS1, and COL15A1, whereas cancellous-associated proteins included FN1, PRG4, and the fibrinogen chains FGA, FGB, and FGG. A similar pattern was observed in PHT, where cortical-associated proteins again included SPARC, BGN, DCN, OMD, MMP2, MMP14, and THBS1, while cancellous-associated proteins were enriched for FN1, FGA, FGB, FGG, C4BPA, MPO, and EPX. In PGI, the list of significantly differential proteins was shorter, but cortical-associated proteins such as SPARC, FGFR1, THBS1, BGN, and ALPL, and cancellous-associated proteins including FGA, FGB, FGG, FN1, MPO, and EPX remained detectable.

### 3.4. Functional Network Analysis Highlights Divergent Biological Pathways

**Figure 4:**
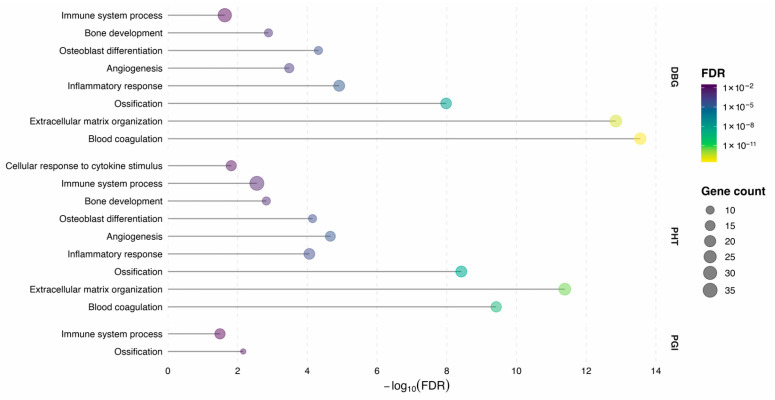
**Functional enrichment of biological processes associated with bone remodeling**. STRING-based Gene Ontology (GO) enrichment analysis was performed separately for each processing condition (DBG, PHT, PGI) using proteins with LFQ abundance above the 70th percentile. Selected GO biological processes relevant to bone repair and remodeling are shown. Enrichment strength is represented by −log10(FDR), point size indicates the number of proteins assigned to each GO term, and point color reflects the corresponding FDR value. GO terms are ordered by the maximal enrichment strength observed across processing conditions.

STRING-based functional enrichment analysis of proteins above the 70th percentile showed that both bone source and processing method were associated with distinct protein abundance patterns (Figure 4). Biological processes representing key phases of bone remodeling were selected for visualization. These included blood coagulation (GO:0007596), cytokine stimulus (GO:0071345), inflammatory response (GO:0006954), immune system process (GO:0002376), angiogenesis (GO:0001525), extracellular matrix organization (GO:0030198), osteoblast differentiation (GO:0001649), ossification (GO:0001503), and bone development (GO:0060348).

Enrichment strength differed across processing steps, as reflected by variation in false discovery rates (FDRs) and the number of contributing proteins within individual GO terms (Figure 4). While some biological processes were detected consistently across multiple processing conditions, others were enriched predominantly in specific steps, indicating condition-dependent representation of functional pathways.

**Figure 5:**
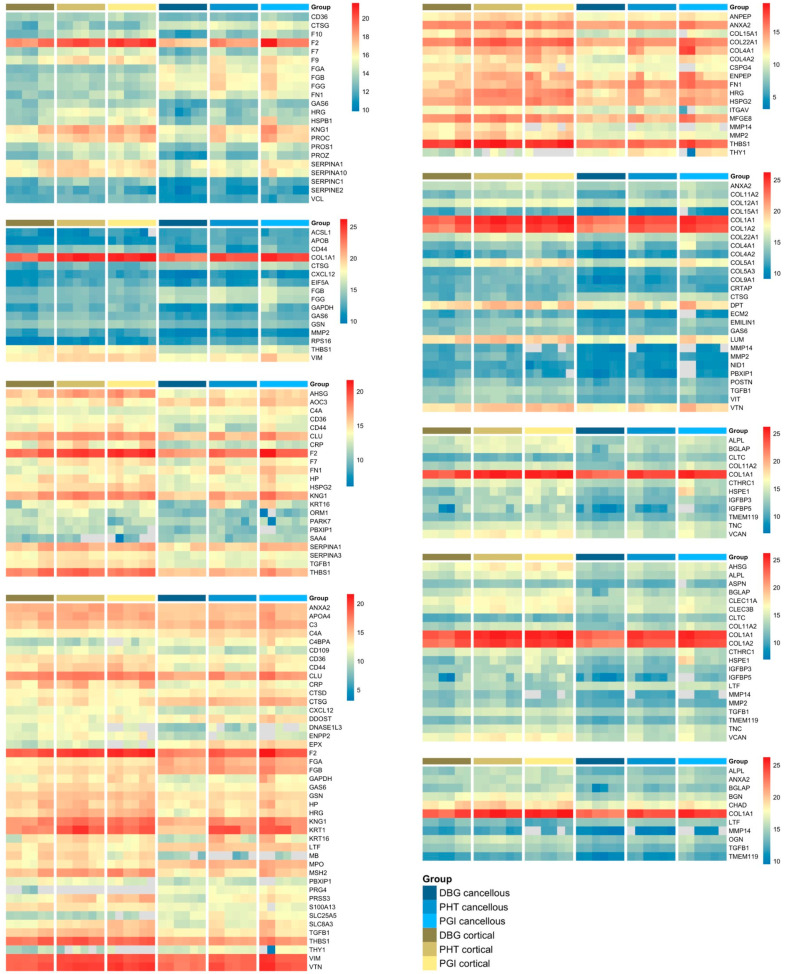
**Heatmap visualization of protein abundance profiles in cortical and cancellous allograft samples under different processing conditions.** Mean label-free quantification (LFQ) intensities from technical HPLC–MS replicates of pooled material are shown for proteins assigned to the selected functional categories. Samples are grouped by allograft type (cortical or cancellous) and processing condition (DBG, PHT, PGI). Cortical samples generally show higher and more uniform signal intensities for structural extracellular matrix proteins, whereas cancellous samples show greater variability and relatively higher levels of inflammatory- and plasma-associated proteins. Color gradients represent relative LFQ intensity from low (blue) to high (red). Each heatmap was scaled independently to visualize within-panel abundance variation across protein groups with different dynamic ranges. Therefore, color intensity should not be compared quantitatively between panels.

Proteins associated with the selected GO terms were subsequently merged across processing steps for heatmap visualization, enabling an integrated comparison of protein abundance patterns across bone types and processing conditions (Figure 5). In cancellous bone, the protein patterns are associated with blood coagulation, cytokine stimulus, inflammatory response, and immune system processes. In contrast, cortical bone exhibited a higher relative abundance of proteins related to extracellular matrix organization, osteoblast differentiation, ossification, and bone development. Processing influenced protein profiles in both bone types. Most proteins exhibited reduced extremes and a more narrow dynamic range after processing, resulting in more homogenized intensity patterns for PHT and PGI compared to DBG. However, a subset of proteins, including collagen chains (COL1A1, COL1A2), prothrombin (F2), vimentin (VIM), and thrombospondin (THBS1), showed an increase in extractable abundance across the processing steps. Despite these shifts, structural and matrix-associated proteins remained detectable under all processing conditions.

### 3.5. Redundancy Analysis of STRING Clusters Illustrates Group-Specific Protein Distributions

**Figure 6:**
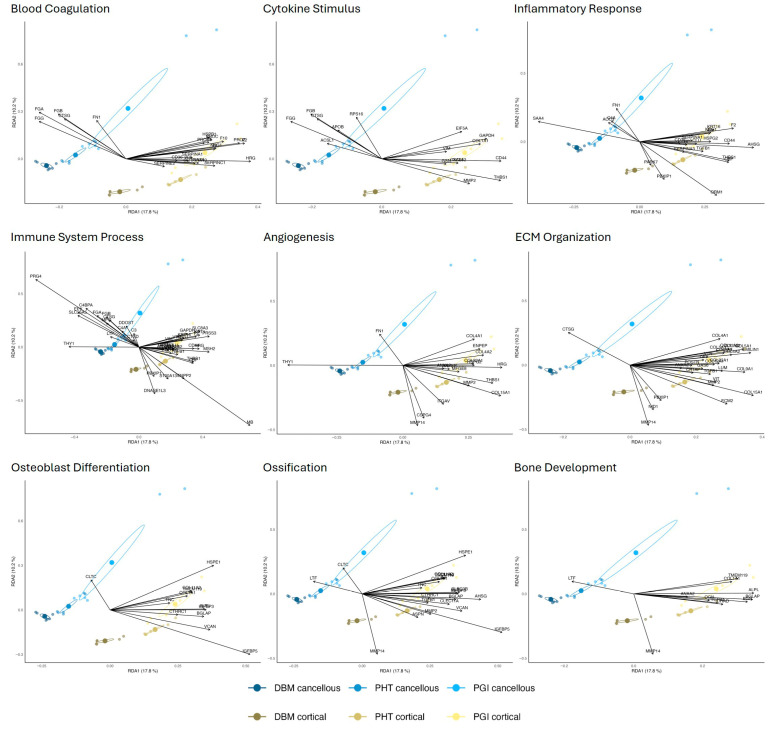
**Projection of STRING-derived protein clusters into the redundancy analysis (RDA) space.** Each panel shows the proteins belonging to one selected STRING/GO functional category, projected onto the RDA ordination derived from the complete proteomic dataset. The same RDA axes as in Figure 1 were retained to enable direct comparison with the full proteome structure. Cortical samples are shown in green and cancellous samples in blue. Processing conditions (DBG, PHT, PGI) are indicated. The clustering patterns illustrate how functionally related protein subsets contribute to the overall separation by bone type and processing stage.

To further assess how the proteins in the GO-term panels contribute to variance in the dataset, they were projected into the previously computed RDA space (Figure 1). Each panel represents the same biological processes as shown in the heatmap categories in Figure 5, and the RDA axes were retained to allow direct comparison with the full proteome ordination (Figure 6). This projection provides an overview of how these functional subsets map onto the dominant trends driven by bone type and processing. Proteins aligning with the cancellous region of the ordination space predominantly corresponded to blood coagulation, cytokine stimulus, inflammatory response, and immune system processes. Among these, the immune system process group showed the strongest cancellous-associated signal, with its projected proteins extending furthest toward that domain. Processes related to extracellular matrix organization, osteoblast differentiation, ossification, and bone development were shifted more strongly in the cortical direction. In these panels, cortical proteins formed tighter and more clearly separated clusters. The degree of group dispersion also varied across the GO terms. Early-phase inflammatory and immune groups showed greater within-group heterogeneity. In contrast, late-phase structural groups showed narrower distributions, particularly towards cortical PHT and PGI samples.

## 4. Discussion

### 4.1. Bone-Type-Specific Signatures and Biological Rationale

This study demonstrates that cortical and cancellous allografts retain distinct extracellular matrix protein profiles despite standardized processing for clinical use. Rather than yielding a fully homogenized ECM composition, processing preserves detectable bone type-specific differences. Cancellous grafts were enriched in proteins associated with inflammatory, coagulative, and immune-related processes, whereas cortical grafts showed higher abundance of structural and matrix-organizing proteins (Figure 5). These findings suggest that both graft sources exhibit distinct profiles of extractable extracellular matrix proteins and may be relevant to biological performance after implantation. However, the present study does not directly assess clinical outcomes and should not be interpreted as evidence of graft superiority for specific indications. The proteomic distinction between the two bone types is consistent with their native architecture and remodeling context. Cancellous bone is highly porous, strongly vascularized, and closely associated with marrow-rich compartments, whereas cortical bone is dense, lamellar, and mechanically load-bearing. These structural and biological differences are likely to influence the extractable ECM-associated proteome. The observed enrichment of immune-modulatory proteins in cancellous grafts is consistent with their close association with bone marrow, while cortical bone shows relative enrichment of highly abundant collagenous and mechanically stabilizing matrix components. This observation aligns with paleoproteomic studies, which show that cortical bone generally preserves larger and less degraded proteomes than cancellous bone [18].

### 4.2. Functional ECM Proteomic Differences Reflect Distinct Phases of Graft Incorporation

The functional annotation of the retained ECM proteins supports differences in the extractable proteomic profiles of processed cortical and cancellous allografts during graft incorporation. The native three-dimensional architecture and matrix density likely influence protein preservation and protein accessibility and therefore shape the composition of the extractable ECM proteome. Functional enrichment analysis performed separately for each processing condition, together with STRING-based clustering, supports this interpretation.

Cancellous grafts were enriched in proteins associated with blood coagulation, inflammatory response, cytokine stimulus, and immune system processes. Fibrinogen chains (FGA, FGB, FGG), fibronectin (FN1), and thrombospondin-1 (THBS1) have been associated with early phases of bone repair, including hematoma formation, fibrin clot generation, immune cell recruitment, and angiogenesis [32,49,50]. These findings are therefore consistent with a molecular profile more closely associated with early inflammatory and fibrovascular stages. In contrast, cortical grafts were relatively enriched in proteins related to extracellular matrix organization, osteoblast differentiation, ossification, and bone development. Decorin (DCN), biglycan (BGN), MMP-2, and MMP-14 are involved in collagen fibrillogenesis and matrix maturation [51,52]. The data reflect a proteomic pattern that corresponds with clinical practice, as cortical allografts provide biomechanical support and a scaffold for slow replacement over time [53]. Overall, these results support distinct ECM-associated proteomic patterns between processed cortical and cancellous allografts.

### 4.3. Impact of Processing for Clinical Use on ECM Protein Retention and Extractability

Beyond these inherent biological differences, allograft processing also influences ECM proteome composition and availability upon protein extraction. Allografts often require many months for full incorporation, which is consistent with a matrix-rich profile rather than a cytokine-rich and pro-inflammatory healing pattern [54]. The projection of STRING-defined GO-term groups into RDA space further suggests that proteins associated with early inflammatory and immune-related categories map preferentially toward the cancellous region, whereas proteins linked to structural and late-stage matrix-related categories align more strongly with the cortical direction. These patterns may indicate source-associated differences in the extractable protein milieu that are relevant to graft incorporation. However, the present study is descriptive and proteomic in nature and does not establish causal biological differences between graft types.

Processing-related effects were also evident. Most proteins exhibited reduced extremes and narrowing of the dynamic range with progressive processing, which likely reflects partial denaturation and/or altered extractability, particularly in the PGI samples. Previous studies indicate that gamma irradiation and other sterilization methods can reduce the osteoinductive potential of allografts [55]. Our data are consistent with reduced proteomic complexity after progressive processing, but they do not directly measure osteoinductive function.

Interestingly, certain proteins (e.g., COL1A1, COL1A2, F2, VIM, THBS1) exhibited increased extractable abundance throughout processing (Figure 2). This finding suggests that processing does not uniformly reduce protein detectability but may instead enhance extraction of specific ECM-associated or cytoskeletal proteins. The higher relative abundance of extractable proteins in cortical samples may result from the distinct three-dimensional architecture of the two bone types. Dense cortical lamellae may protect embedded ECM proteins from extraction and degradation, thereby favoring retention even under harsh processing. In contrast, the cancellous trabecular network, high marrow content, and large surface area may enrich immune- and plasma-derived proteins while also rendering them more susceptible to removal or alteration during processing. It should be noted that the present analysis captures ECM-associated proteins that are extractable and detectable rather than absolute protein retention within the graft material. Differences in extractability may therefore reflect both preservation and altered accessibility of matrix components induced by processing.

### 4.4. Proteome-Based Implications for Allograft Standardization and Quality Control

In addition to their biological relevance, proteomic profiles may provide a framework for supporting standardization and quality control of bone allograft products. Proteome-based analyses offer an objective molecular measure of tissue-specific composition and processing-related modifications, enabling assessment of product consistency and matrix quality across batches. Such molecular characterization may complement existing physical and biochemical quality criteria by capturing processing-induced changes that are not detectable by conventional assays.

### 4.5. Study Limitations

Several limitations should be considered. First, the analyses were performed on pooled donor manufacturing material, and the reported replicates are technical replicates rather than donor-level biological replicates. The study therefore does not permit assessment of interindividual variability, including possible effects of sex, age, or donor-specific background. Second, donor pool sizes differed markedly between groups and processing stages, with substantially larger pools for cancellous than cortical material and a particularly small cortical PGI donor pool. Third, the anatomical source was defined at a broad level, with cortical material derived from the femoral diaphysis and cancellous material from the femoral head. Fourth, parts of the regulated clinical manufacturing workflow cannot be disclosed in detail, which limits strict methodological reproducibility. Finally, the present study does not include cell-based functional testing or mechanistic validation and therefore does not establish causal biological differences between graft types. The identified proteomic differences provide candidate matrix signatures for future functional validation. Accordingly, the present findings reflect pooled manufacturing-lot proteomic signatures rather than donor-resolved biological variability and should therefore be generalized with caution. A rigorous functional comparison would require a prespecified cell-based design including responder cell type, matrix presentation format, and normalization strategy for processed graft material, which was beyond the scope of the present proteomic study.

## 5. Conclusions

In summary, processed cortical and cancellous allografts retained distinguishable patterns in the extractable ECM-associated proteome. Cancellous-derived products showed relative enrichment of proteins linked to coagulation, inflammatory, and immune-related processes, whereas cortical-derived products were relatively enriched in structural and matrix-organization-associated proteins. These source-associated signatures persisted despite progressive processing, although the number of strongly differential proteins decreased. Given the use of pooled donor material, unequal donor pool sizes, and the lack of functional validation, these observations should be interpreted as descriptive proteomic signatures that warrant further donor-resolved and cell-based investigation.

## Figures and Tables

**Table 1:** **Categorization of detected ECM-associated proteins and signaling molecules according to their reported roles in bone repair phases.** Proteins and growth factors were grouped based on their predominant biological functions during the inflammatory, repair/fibrovascular, and remodeling phases of bone regeneration, as well as cross-phase regulatory roles. The listed molecules include ECM structural components, cytokine-binding and signaling proteins, angiogenic mediators, and enzymes.

Phase	Function	Proteins/Molecules	Remarks
Inflammatory Phase	Cytokine binding/signaling	**Biglycan**, **Decorin**	Bind TLR2/4 → immune activation; sequestration of TGF-β [27,28,29]
	Pro-inflammatory mediators	**TGF-β1**, TNF-α, IL-1β, IL-6, **PDGF**, FGF-2	Released in response to tissue damage and orchestrate the early inflammatory response [30,31]
	Cell adhesion & migration	**Fibronectin**, **Thrombospondin-1**, **Laminin**	Promote adhesion and migration of immune and MSCs [32,33]
Repair/Fibro-Vascular Phase	Angiogenesis	VEGF, FGF-2, **heparan-sulfate proteoglycans (HSPG)**	HSPG stabilize VEGF/FGF gradients [34,35,36]
	Matrix assembly (structure)	**Collagen I**, **III**, **V**; **Fibronectin**	Collagens form the scaffold of the soft callus [37,38]
	Cell proliferation & differentiation	**TGF-β1**, BMP-2/4/7, Wnt5a	Activation of osteogenic pathways (e.g., SMAD, Wnt) [39,40]
Remodeling Phase	Progenitor recruitment	SDF-1, **Osteopontin**	Promote MSC recruitment and homing [31,41]
	Osteogenesis & mineralization	**Collagen I**, **Osteocalcin**, **Osteonectin (SPARC)**	Osteocalcin & SPARC participate in mineral binding [31]
	Modulation of mineralization	**Biglycan**, **Decorin**, **Matrix Gla protein**	Collagen fibril organization and osteoblast responses, influencing matrix mineralization [42,43]
	ECM remodeling	**MMP-2**, **MMP-9**, Cathepsin K, **LOX**	Collagen degradation and cross-linking [44]
Cross-Phase Functions	Growth-factor binding	**Heparan sulfate**, Perlecan, **Biglycan**, **Decorin**	Regulate the bioavailability of TGF-β (decorin/biglycan) and heparin-binding growth factors such as FGF and PDGF (HS/perlecan) [45,46]
	Integrin–ligand interactions	**Fibronectin**, **Laminin**	Promote adhesion and lineage signaling via integrins [47,48]

Only proteins detected in the present study are highlighted in bold; non-bold entries are included for functional context and were not detected.

## Data Availability

The mass spectrometry proteomics data generated in this study have been deposited to the ProteomeXchange Consortium via the PRIDE partner repository, a public repository for MS-based proteomics datasets. The dataset is accessible with the identifier PXD075611.

## Supplementary Materials

The following supporting information can be downloaded at: https://www.mdpi.com/article/10.3390/cells15090842/s1, Figure S1: Schematic illustration of the anatomical source regions used for cortical and cancellous allograft preparation. Table S1: Significantly differential proteins between cortical and cancellous allografts in DBG, PHT, and PGI samples.

## Abbreviations

The following abbreviations are used in this manuscript:

ABC	Ammonium bicarbonate
ACN	Acetonitrile
BCA	Bicinchoninic acid
BGN	Biglycan
BMP	Bone morphogenetic protein
BSA	Bovine serum albumin
DBG	Demineralized bone granules
DBM	Demineralized bone matrix
DCN	Decorin
DIA	Data-independent acquisition
ECM	Extracellular matrix
EDQM	European Directorate for the Quality of Medicines & HealthCare
FA	Formic acid
FAIMS	Field asymmetric ion mobility spectrometry
FDR	False discovery rate
FGF	Fibroblast growth factor
FN1	Fibronectin 1
GBVO	Gewebebankenverordnung
GO	Gene Ontology
GPF	Gas-phase fractionation
HCD	Higher-energy collisional dissociation
HPLC–MS	High-performance liquid chromatography with mass spectrometry
HSPG	Heparan sulfate proteoglycan
IL	Interleukin
LFQ	Label-free quantification
LOX	Lysyl oxidase
MMP	Matrix metalloproteinase
MSC	Mesenchymal stromal/stem cell(s)
NCE	Normalized collision energy
OMD	Osteomodulin
PGI	Putty gamma-irradiated
PHT	Putty heat-treated
PRIDE	PRoteomics IDEntifications database
RDA	Redundancy analysis
SMAD	Small mothers against decapentaplegic
STRING	Search Tool for the Retrieval of Interacting Genes/Proteins
TGF	Transforming growth factor
THBS1	Thrombospondin-1
TNF	Tumor necrosis factor
VEGF	Vascular endothelial growth factor
WFI	Water-for-injection
