# Supplementary figures and images for: Distinct Extracellular Matrix Protein Signatures of Cortical and Cancellous Bone Allografts Following Processing for Clinical Use
Source: Cells. 2026 May 4;15(9):842. doi: 10.3390/cells15090842 (PMC13163088; doi:10.3390/cells15090842)

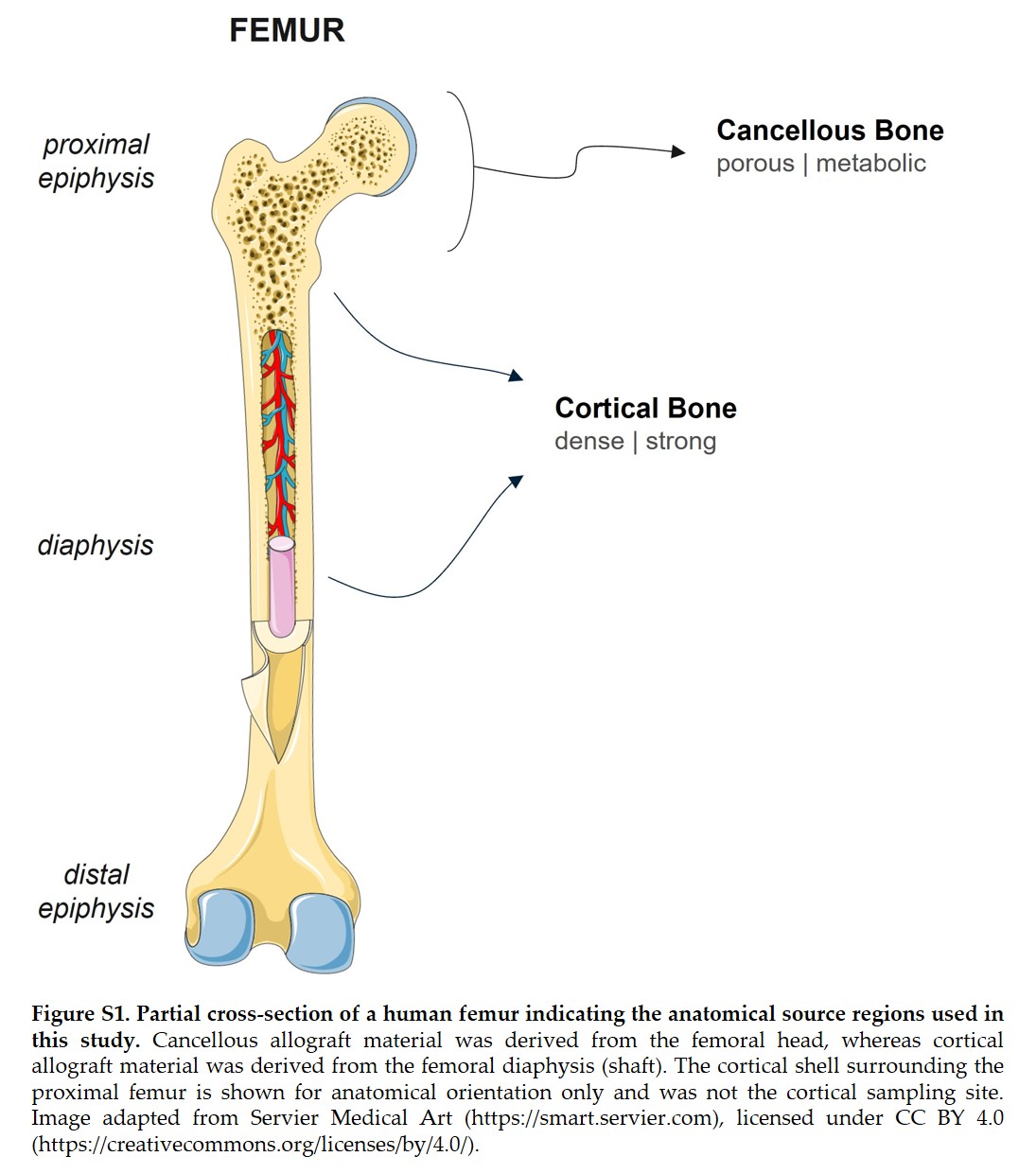

Supplement: Supplementary file 1 [file cells-15-00842-s001.zip › Figure S1 Schematic illustration of the anatomical source regions used for cortical and cancellous allograft preparation..jpg]
